# Bone Mineral Density is Negatively Associated with Risk of All-Cause and Cardiovascular Mortality among Adults with Type 2 Diabetes Mellitus: A Cross-sectional Study of the NHANES 2005–2010, 2013–2014

**DOI:** 10.31083/j.rcm2512434

**Published:** 2024-12-11

**Authors:** Haipeng Li, Baolong Wang, Dongshuo Xu, Jialu Zhang, Changhui Wang

**Affiliations:** ^1^Department of Cardiology, The First Affiliated Hospital of Anhui Medical University, 230022 Hefei, Anhui, China

**Keywords:** bone mineral density, osteoporosis, type 2 diabetes, cardiovascular mortality, all-cause mortality, NHANES database

## Abstract

**Background:**

With ageing and lifestyle changes, the coexistence of osteoporosis and type 2 diabetes (T2DM) is becoming more common, which greatly increases patient disability and mortality. However, the association of low bone mineral density (BMD) with cardiovascular disease (CVD) and all-cause mortality in T2DM patients have not been conclusively established.

**Methods:**

Using the National Health and Nutrition Examination Survey (NHANES) to obtain a nationally representative sample of the US population, we sought to determine the independent and incremental value of low BMD, particularly in patients with osteoporosis in assessing all-cause and CVD mortality in adults with T2DM.

**Results:**

We demonstrated that increased BMD was significantly related to decreased mortality from all-causes and CVDs among US adults with T2DM. In addition, we found that, after multivariate adjustment, osteoporosis and osteopenia were independently associated with an increased risk of all-cause and CVD mortality in T2DM patients at long-term follow-up.

**Conclusions:**

The clinical diagnosis of osteopenia or osteoporosis in adults with T2DM provides independent prognostic value for CVD and all-cause mortality.

## 1. Introduction

Diabetes mellitus is a collection of common metabolic endocrine diseases characterized by abnormalities of glucose and fat metabolism, as well as elevated plasma glucose [1]. According to the World Health Organization, more than 463 million people worldwide had diabetes in 2015, and this figure is expected to double by 2040 as the population ages [2]. Type 2 diabetes mellitus (T2DM) is the most common type of diabetes, accounting for more than 90% of the diabetic population and affecting nearly 22% of older adults in the United States [3].

Bone mineral density (BMD) is a quantifiable measure of bone mass and strength, determined by the mineral content in bone tissue [4]. It can be used for the diagnosis of osteoporosis, to predict the risk of fractures, and to assess the efficacy of drug therapy. Osteoporosis is a condition that affects the entire skeletal system, causing reduced bone density and deterioration of bone structure, leading to increased vulnerability to fractures [5]. With an ageing global population and changing lifestyle habits, the increasing incidence and associated socio-economic burden of osteoporosis worldwide have become a critical public health issue [6]. It is estimated that 10.2 million Americans over the age of 50 suffered from osteoporosis and 43.4 million suffered from osteopenia in 2010 [7].

T2DM and osteoporosis have similar risk factors and common pathophysiological characteristics. The two often coexist, which can exacerbate each other, thus worsening the prognosis of patients and increasing mortality. T2DM is known to affect the metabolism of sugar, fat and protein, as well as causing imbalances in calcium, phosphorus and magnesium, and subsequently promoting a range of complications such as neuropathy, cardiovascular disease (CVD), peripheral vascular disease, retinopathy and metabolic bone disease [8, 9]. Certain medications used to control high blood sugar may exacerbate bone complications. There is also an association between diabetic complications and risk for falls and subsequent fractures in osteoporosis [10]. As a result, patients with T2DM are at a high risk for osteoporosis, with approximately one-third having combined osteoporosis [11]. Brittle fractures caused by osteoporosis have become an important cause of death and disability in patients with T2DM [12].

Although previous studies have looked into the relationship between osteoporosis and chronic diseases such as CVD and cancer, the majority of these studies have focused on women who have had osteoporotic fractures, postmenopausal women, or the elderly, with a particular emphasis on the relationship between osteoporosis and the incidence of CVD [13, 14, 15]. The coexistence of osteoporosis and T2DM is becoming more common with age and lifestyle changes, which influences patients’ disability and mortality [16]. Nevertheless, the associations of a reduced BMD value with CVD and all-cause mortality in T2DM patients have not been definitively proven. Neglecting the potential link between these diseases could have serious consequences. It is crucial to delve into further research in order to ascertain the precise relationship between these risk factors, allowing for the implementation of effective preventive measures to reduce disease risks. As a result, a large cohort research study involving this specific demographic is required, as well as a more in-depth review of the influence on CVD and mortality risk.

In this study, we used the National Health and Nutrition Examination Survey (NHANES) to evaluate the relationship between BMD and the risk of CVD and all-cause death in the US population with T2DM.

## 2. Methods

### 2.1 Study Population

The National Centre for Health Statistics of the Centers for Disease Control and Prevention conducts the NHANES, a periodic cross-sectional sampling survey, on a nationally representative sample of the non-institutionalized civilian population in the United States. Participants were asked to complete standardized questionnaires about their demographic and socioeconomic characteristics, health-related activities, and health problems. Throughout the study’s recruitment phase, trained interviewers distributed and collected questionnaires. Physical exams and laboratory tests were carried out by trained medical specialists at mobile examination facilities. The nature of the sampling processes and analytical criteria have already been made public. At enrollment, all NHANES participants submitted informed written consent, and the study methods were approved by the National Centre for Health Statistics’ Institutional Review Board.

**Fig. 1.**
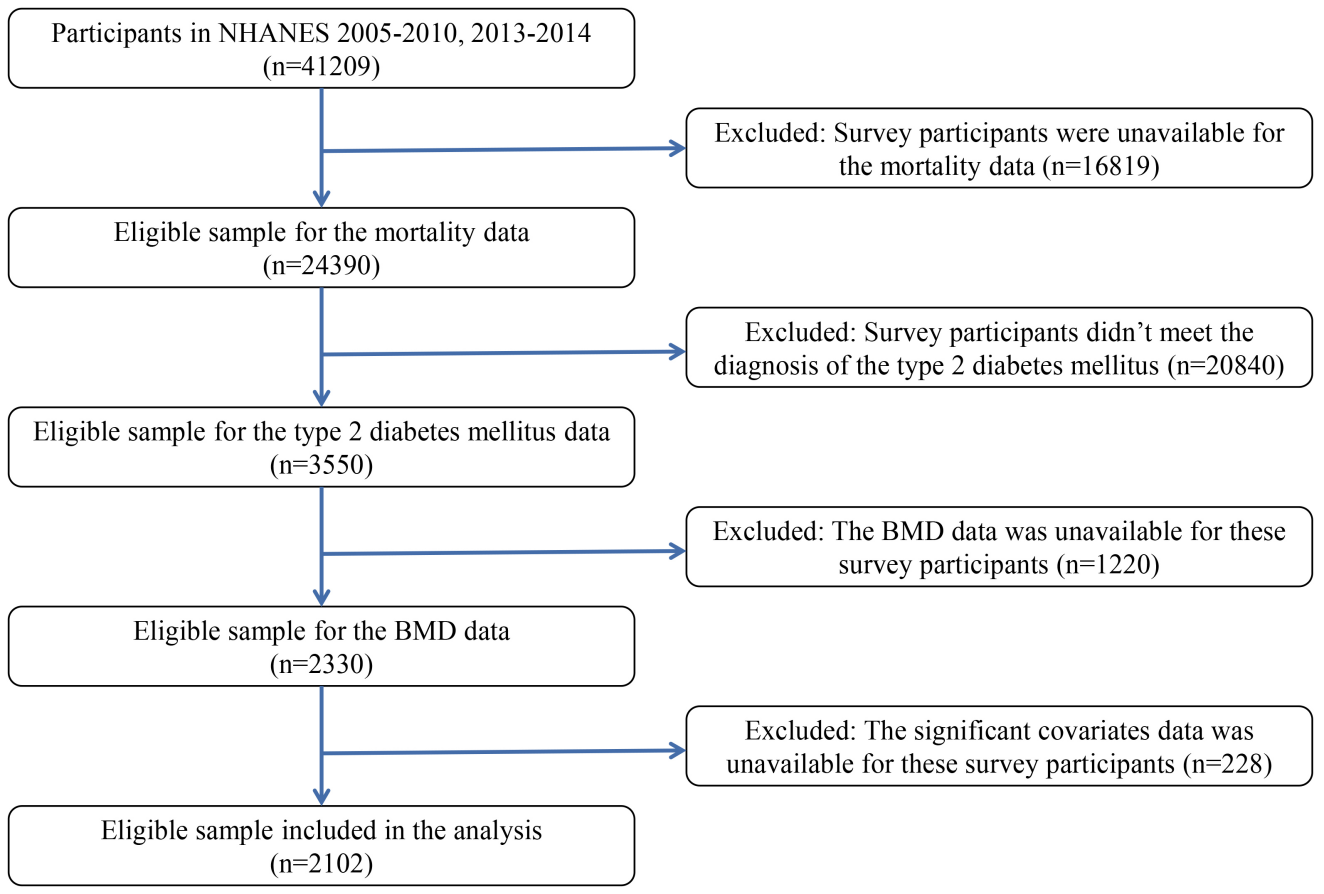
**The flowchart for the selection of study participants**. Abbreviations: BMD, bone mineral density; NHANES, National Health and Nutrition Examination Survey.

This research involved individuals aged 20 years and older with T2DM from four rounds of NHANES III conducted between 2005 and 2010, as well as 2013 and 2014. NHANES III (2011–2012) and other cycles did not provide data on total femur BMD and femoral neck BMD, leading to their exclusion from our analysis. We excluded participants without mortality data (n = 16,819). We identified T2DM by determining if participants met the American Diabetes Association criteria, which included self-reported physician diagnosis of diabetes, use of oral glucose-lowering medications or insulin, and fasting plasma glucose levels of 126 mg/dL or higher, 75 g oral glucose tolerance test results of 200 mg/dL or higher (to convert glucose to mmol/L, multiply by 0.0555), or hemoglobin A1c (HbA1c) levels of 6.5% or higher (48 mmol/mol) (to convert HbA1c percentage of total hemoglobin to a proportion of total hemoglobin, multiply by 0.01). A total of 3550 participants met the diagnostic criteria for diabetes. Participants with T2DM were included after excluding participants who were without BMD data (n = 1220), or were without significant covariates data (n = 228), 2102. Fig. 1 displays the flowchart outlining the process for selecting study participants.

### 2.2 BMD Measurement and Definition of Osteoporosis

All individuals included in the final analysis had their BMD was assessed using Hologic QDR-4500A fan-beam densitometers (Hologic; Bedford, MA, USA) by qualified radiology technologists. We utilized Hologic APEX (version 4.0, Hologic, Inc., Marlborough, MA, USA) to examine the results of all DXA (dual-energy X-ray absorptiometry) scans. In this study, the mean femoral BMDs of non-Hispanic white men and women aged 20–29 from NHANES III were utilized as reference values for men and women, respectively (see Looker *et al*. [17] for details). All subjects were classified into normal, osteopenia, and osteoporosis categories based on total femur (TF) and femoral neck (FN) BMD. Osteoporosis was defined as BMD (total femur or femoral neck) below the reference value [18]. The reference values used to establish a diagnosis of normal, osteopenia, or osteoporosis are shown in **Supplementary Table 1**.

### 2.3 Ascertaining Mortality

Connecting the cohort database to the National Death Index until December 31, 2015 was used to determine mortality. The overall mortality rate included deaths from any cause. We used categories I00–I09, I11–13, I20–I51, and I60–I69 from the Tenth Revision of the International Statistical Classification of Diseases and Related Health Problems to determine CVD mortality.

### 2.4 Assessment of Covariates

Trained interviewers handed out questionnaires to participants during the NHANES interview to gather data on demographics (such as age, gender, race and ethnicity, educational attainment, and poverty income ratio [PIR]), smoking habits, drinking behavior, physical exercise, and waist size. Body mass index (BMI) was determined by dividing the weight by the square of the height in kilograms per square meter. BMI was categorized into four groups based on guidelines from the World Health Organization: (i) under 18.5 kg/m^2^; (ii) 18.5 to 25 kg/m^2^; (iii) 25 to 30 kg/m^2^; (iv) 30 kg/m^2^ or higher. Race and ethnicity were classified as non-Hispanic White, non-Hispanic Black, Mexican American, other Hispanic, and other/multiracial. The PIR for the family’s income level was classified into three groups: ≤1.0, 1.0–3.0, and >3.0. Levels of educational achievement were categorized as below 9th grade, 9th–11th grade, completion of high school or general educational development (GED), completion of some college or an associate’s degree, and completion of college or higher. Alcohol intake was categorized as consuming 1–5 drinks per month, 5–10 drinks per month, or abstaining from alcohol. Participants’ smoking status was classified into three groups: never smoked (less than 100 cigarettes in their lifetime), former smokers (over 100 cigarettes in their lifetime but currently quit), and current smokers (over 100 cigarettes in their lifetime and still smoking). Physical activity was measured as the weekly minutes of moderate and vigorous activities multiplied by the metabolic equivalent (MET) level and divided into three categories: no physical activity (without regular physical activity, MET-minutes/week = 0), low physical activity (0 < MET-minutes/week < 600), and high physical activity (>600 MET-minutes/week) [19].

The blood samples were frozen at a temperature of –20 °C before being sent to the National Centre for Environmental Health for examination. This includes high density lipoprotein cholesterol (HDL-C), total cholesterol (TC), total triglycerides (TG), creatinine (Cre), blood urea nitrogen (BUN), uric acid (UA), total serum calcium, and creatinine. The NHANES website [20] provided detailed descriptions of the laboratory procedures.

### 2.5 Statistical Analysis

Due to the intricate sampling design of NHANES, sample weights, clustering, and stratification were included in all analyses conducted in this study. The person-years for each participant were determined starting from the recruitment date until either the date of death or the end of follow-up on December 31, 2015, whichever came first. Participants were categorized into three groups based on their BMD (total femur or femoral neck): normal bone mass, osteopenia, and osteoporosis. Baseline characteristics were reported as weighted median with quartiles for continuous variables and as frequency with weighted percentages for categorical variables. Differences among groups were compared using analysis of variance (ANOVA) for continuous variables and the chi-square test for categorical variables. Kaplan-Meier analysis was utilized to assess the disparities in all-cause mortality and CVD mortality among the various participant groups. Hazard ratios (HRs) and 95% confidence interval (CI) were calculated using multivariable Cox proportional hazards regression models to examine the relationship between BMD and the likelihood of CVD and all-cause mortality in American adults diagnosed with T2DM. Three multivariable models were constructed. A restricted cubic spline analysis was conducted using 4 knots at the 5th, 35th, 65th, and 95th percentiles to explore the non-linear relationship between BMD and the risk of CVD and all-cause mortality in American adults with T2DM. The normal group was used as a reference, and values between the first and 95th percentiles were considered to reduce the impact of outliers. The analysis was stratified by various factors and underwent sensitivity tests. R studio (version 4.1.3, Posit, 250 Northern Avenue, Suite 420, Boston, MA, USA) was used for all analyses, with a significance threshold of *p* < 0.05 set for statistical significance on both sides.

## 3. Results

### 3.1 Baseline Characteristics

Our analysis included 2102 T2DM participants aged 20 and older, including 49 patients with osteoporosis, 506 patients with osteopenia, and 1547 patients with normal bone mass. The baseline data of individuals with osteoporosis, osteopenia, and normal bone mass is presented in Table 1. Individuals diagnosed with osteoporosis and osteopenia tended to be female, of non-Hispanic white descent, and older compared to individuals with normal bone density. They were also more likely to have a lower BMI, lower levels of education, and less physical activity, but they drank less often. Participants with osteopenia and osteoporosis had a thinner waist, higher HDL-C and BUN, lower blood lipid levels, and UA than those with normal bone mass. The median BMD value of the total femur in normal bone mass participants was 1.04 g/cm^2^, and the median BMD value of the femoral neck was 0.86 g/cm^2^. In osteopenia patients, the median BMD value of the entire femur was 0.80 g/cm^2^, while the median BMD value of the femoral neck was 0.66 g/cm^2^. Osteoporosis participants had a median BMD value of 0.61 g/cm^2^ for the entire femur and 0.52 g/cm^2^ for the femoral neck, which was considerably lower than that of normal bone mass participants.

**Table 1.** Baseline characteristics according to the groups of bone mineral density*.

Characteristic		N^1^	Overall	Normal	Osteopenia	Osteoporosis	*p *value^3^
			N = 2102 (100%)^2^	N = 1547 (75%)^2^	N = 506 (23%)^2^	N = 49 (2.0%)^2^	
Gender (n, %)		2102					<0.001
	Female		916 (43.57%)	617 (40.80%)	259 (49.36%)	40 (79.37%)	
	Male		1186 (56.43%)	930 (59.20%)	247 (50.64%)	9 (20.63%)	
Age [years, M (Q1, Q3)]		2102	60 (50, 69)	58 (49, 67)	66 (56, 76)	77 (67, 80)	<0.001
BMI group (n, %)		2102					<0.001
	Underweight (<18.5 kg/m^2^)		6 (0.25%)	1 (0.04%)	5 (0.95%)	0 (0%)	
	Normal weight (18.5 to <25 kg/m^2^)		301 (12.75%)	149 (7.82%)	127 (24.82%)	25 (56.27%)	
	Overweight (25 to <30 kg/m^2^)		709 (30.28%)	475 (27.48%)	215 (38.65%)	19 (36.91%)	
	Obese (30 kg/m^2^ or greater)		1086 (56.72%)	922 (64.66%)	159 (35.58%)	5 (6.82%)	
Race and ethnicity (n, %)		2102					<0.001
	Non-Hispanic White		842 (64.11%)	581 (62.37%)	237 (69.66%)	24 (64.56%)	
	Non-Hispanic Black		492 (13.81%)	408 (15.60%)	74 (8.17%)	10 (12.82%)	
	Mexican American		444 (10.18%)	332 (10.72%)	103 (8.42%)	9 (10.47%)	
	Other Hispanic		196 (5.34%)	142 (5.45%)	52 (5.29%)	2 (1.68%)	
	Other/multiracial		128 (6.56%)	84 (5.86%)	40 (8.46%)	4 (10.47%)	
Family income to poverty ratio (n, %)		2102					0.005
	<1		435 (13.66%)	309 (13.29%)	112 (13.95%)	14 (24.04%)	
	1–3		989 (42.09%)	704 (40.52%)	261 (46.70%)	24 (46.73%)	
	≥3		678 (44.25%)	534 (46.19%)	133 (39.35%)	11 (29.23%)	
Education attainment (n, %)		2102					<0.001
	Less Than 9th Grade		389 (11.26%)	259 (9.99%)	117 (14.58%)	13 (20.14%)	
	9–11th Grade		394 (14.91%)	301 (14.80%)	83 (15.11%)	10 (16.83%)	
	High School Grad/GED		495 (25.15%)	337 (23.12%)	145 (31.40%)	13 (28.05%)	
	Some College or AA degree		516 (29.79%)	417 (32.84%)	93 (21.20%)	6 (16.34%)	
	College Graduate or above		308 (18.89%)	233 (19.25%)	68 (17.71%)	7 (18.64%)	
Alcohol consumption (n, %)		2102					<0.001
	1–5 drinks/month		1050 (52.18%)	806 (54.62%)	230 (45.65%)	14 (38.05%)	
	5–10 drinks/month		101 (5.75%)	72 (5.44%)	29 (7.21%)	0 (0%)	
	10+ drinks/month		196 (9.63%)	158 (10.70%)	36 (6.74%)	2 (3.44%)	
	Non-drinker		755 (32.44%)	511 (29.24%)	211 (40.40%)	33 (58.51%)	
Smoking status (n, %)		2102					0.089
	Current smoker		348 (15.34%)	248 (15.09%)	88 (15.05%)	12 (28.31%)	
	Former smoker		752 (37.08%)	541 (36.44%)	198 (40.65%)	13 (19.31%)	
	Never smoker		1002 (47.58%)	758 (48.47%)	220 (44.30%)	24 (52.38%)	
Physical activity (n, %)		2102					<0.001
	High physical activity		828 (39.83%)	655 (42.34%)	162 (32.02%)	11 (22.45%)	
	Low physical activity		269 (11.38%)	203 (13.13%)	64 (12.64%)	2 (4.08%)	
	No physical activity		1005 (48.79%)	689 (44.53%)	280 (55.34%)	36 (73.47%)	
Waist [cm, M (Q1, Q3)]		2102	107.80 (98.06, 117.70)	109.97 (101.00, 119.90)	101.96 (92.50, 110.80)	88.28 (82.50, 97.96)	<0.001
HDL-C [mg/dL, M (Q1, Q3)]		2102	45.00 (38.00, 55.00)	45.00 (38.00, 54.00)	46.00 (38.00, 55.00)	57.00 (43.42, 68.99)	<0.001
TC [mg/dL, M (Q1, Q3)]		2102	179.00 (154.00, 213.00)	181.00 (156.00, 214.00)	173.00 (149.00, 208.16)	160.10 (153.00, 220.22)	0.034
TG [mg/dL, M (Q1, Q3)]		2102	157.00 (104.00, 235.00)	159.00 (106.00, 240.00)	150.00 (99.00, 226.64)	119.13 (94.05, 212.79)	0.023
Cre [mg/dL, M (Q1, Q3)]		2102	0.90 (0.75, 1.08)	0.90 (0.74, 1.07)	0.90 (0.76, 1.10)	0.90 (0.81, 1.15)	0.608
BUN [mg/dL, M (Q1, Q3)]		2102	14.00 (11.00, 18.00)	14.00 (11.00, 18.00)	15.00 (11.00, 19.00)	17.28 (15.00, 23.29)	<0.001
UA [mg/dL, M (Q1, Q3)]		2102	5.60 (4.70, 6.60)	5.70 (4.80, 6.60)	5.40 (4.60, 6.50)	5.08 (4.01, 6.04)	0.004
Total serum calcium [mg/dL, M (Q1, Q3)]		2102	9.40 (9.20, 9.70)	9.40 (9.20, 9.70)	9.40 (9.20, 9.70)	9.48 (9.20, 9.70)	0.504
Cotinine [ng/mL, M (Q1, Q3)]		2102	0.04 (0.02, 0.62)	0.04 (0.02, 0.66)	0.04 (0.02, 0.45)	0.03 (0.01, 29.27)	>0.900
Total femur BMD [g/cm^2^, M (Q1, Q3)]		2102	0.99 (0.87, 1.10)	1.04 (0.96, 1.14)	0.80 (0.74, 0.85)	0.61 (0.57, 0.62)	<0.001
Femur neck BMD [g/cm^2^, M (Q1, Q3)]		2102	0.81 (0.71, 0.92)	0.86 (0.79, 0.95)	0.66 (0.61, 0.72)	0.52 (0.48, 0.58)	<0.001
Cardiovascular mortality (n, %)		2102	181 (7.66%)	99 (5.80%)	72 (12.49%)	10 (20.72%)	<0.001
All-cause mortality (n, %)		2102	546 (23.02%)	314 (17.86%)	198 (35.32%)	34 (72.35%)	<0.001

^1^N not Missing (unweighted). ^2^Median (IQR) for continuous; n (%) for categorical. ^3^Wilcoxon rank-sum test for complex survey samples; chi-squared test with Rao & Scott’s second-order correction. *The NHANES used a complex design. Weight was taken into consideration. All data were analyzed based on weighted estimates with sample weights provided by NHANES. Abbreviations: BMI, body mass index; HDL-C, high density lipoprotein cholesterol; TC, total cholesterol; TG, total triglyceride; Cre, creatinine; BUN, blood urea nitrogen; UA, uric acid; BMD, bone mineral density; NHANES, National Health and Nutrition Examination Survey; M,median; Q1, Q3, first quartile, third quartile; Grad, graduate; GED, general educational development; AA, associate of arts; IQR, interquartile range.

### 3.2 Osteopenia, Osteoporosis and Mortality

At the end of the follow-up, we identified 546 all-cause deaths, of which 181 died of CVD. There were 314 all-cause deaths and 99 CVD deaths in the normal bone mass group, 198 all-cause deaths and 72 CVD deaths in the osteopenia group, 34 all-cause deaths and 10 CVD deaths in the osteoporosis group.

**Fig. 2.**
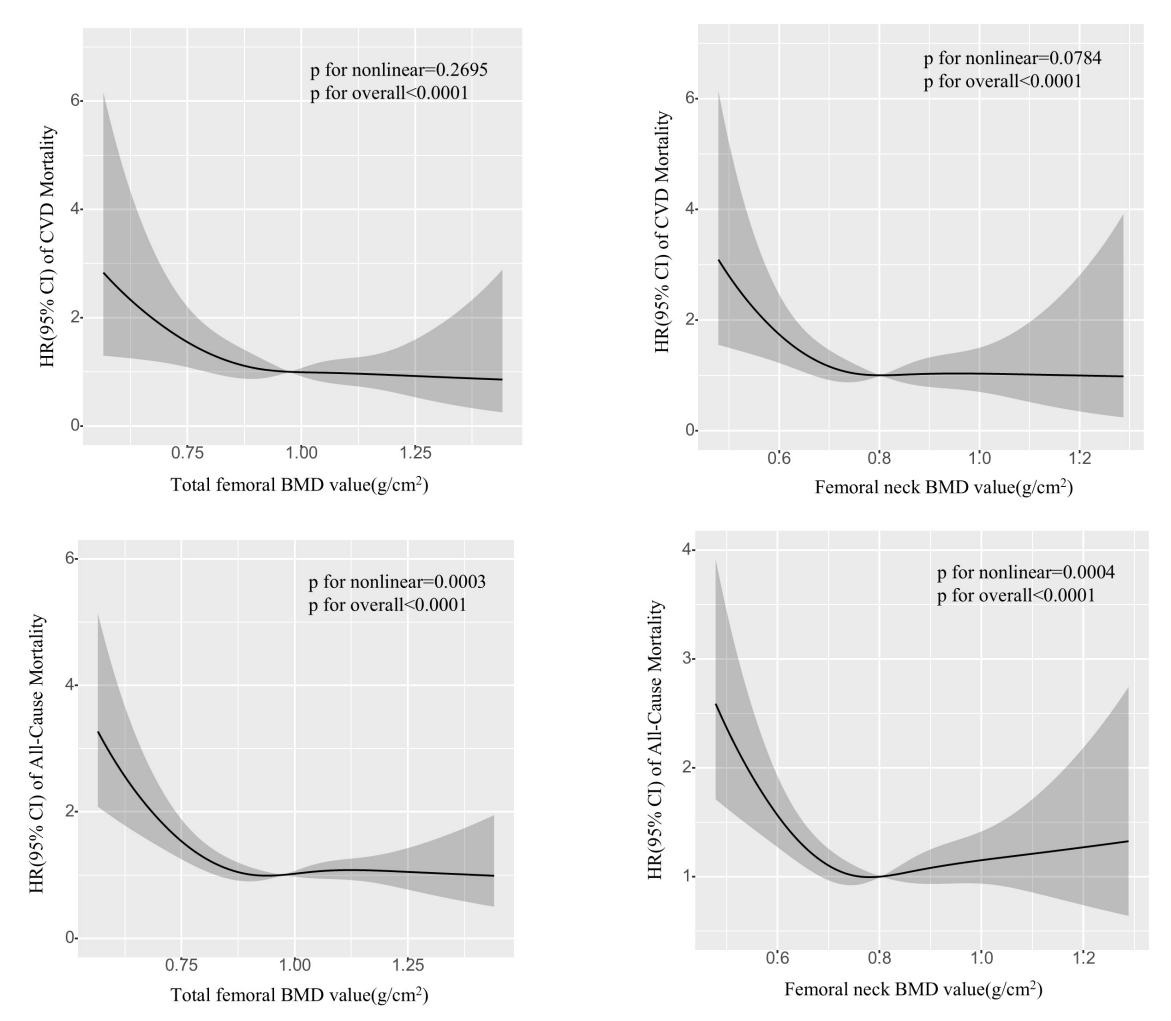
**Potential nonlinear for the value of total femoral and femoral neck BMD with the risk of CVD and all-cause mortality measured by RCS**. The solid line and the frame around it represent the hazard ratios and 95% confidence interval, respectively. Age (continuous), gender (male or female), race and ethnicity (Non-Hispanic White, Non-Hispanic Black, Mexican American, Other Hispanic, Other/multiracial), Education attainment (Less Than 9th Grade, 9–11th Grade, High School Grad/GED, Some College or AA degree, College Graduate or above), Alcohol consumption (1–5 drinks/month, 5–10 drinks/month, 10+ drinks/month, Non-drinker), Smoking status (Current smoker, Former smoker, Never smoker), Physical activity (High physical activity, Low physical activity, No physical activity), Waist, HDL-C, TC, TG, Cre, BUN, UA, Total serum calcium and Cotinine (all continuous) were adjusted. Abbreviations: RCS, restricted cubic spline; HR, hazard ratio; CI, confidence Interval; BMD, bone mineral density; NHANES, National Health and Nutrition Examination Survey; CVD, cardiovascular disease; Grad, graduate; GED, general educational development; HDL-C, high density lipoprotein cholesterol; TC, total cholesterol; TG, total triglyceride; Cre, creatinine; BUN, blood urea nitrogen; UA, uric acid.

Fig. 2 displays the correlation between the combined total femoral BMD value, femoral neck BMD value, and the likelihood of CVD mortality and overall mortality in adults diagnosed with T2DM during the specified time periods of C III (2005–2010, 2013–2014). As the total BMD value or BMD value in the femoral neck decreases in individuals with T2DM, the likelihood of all-cause mortality and CVD mortality rises steadily. Restricted cubic spline (RCS) analysis demonstrated a nearly linear association between the risk of CVD mortality and total femoral BMD value (*p* for nonlinear = 0.2695, *p* for overall <0.0001) and femoral neck BMD value (*p* for non-linearity = 0.0784, *p* for total <0.0001).

**Fig. 3.**
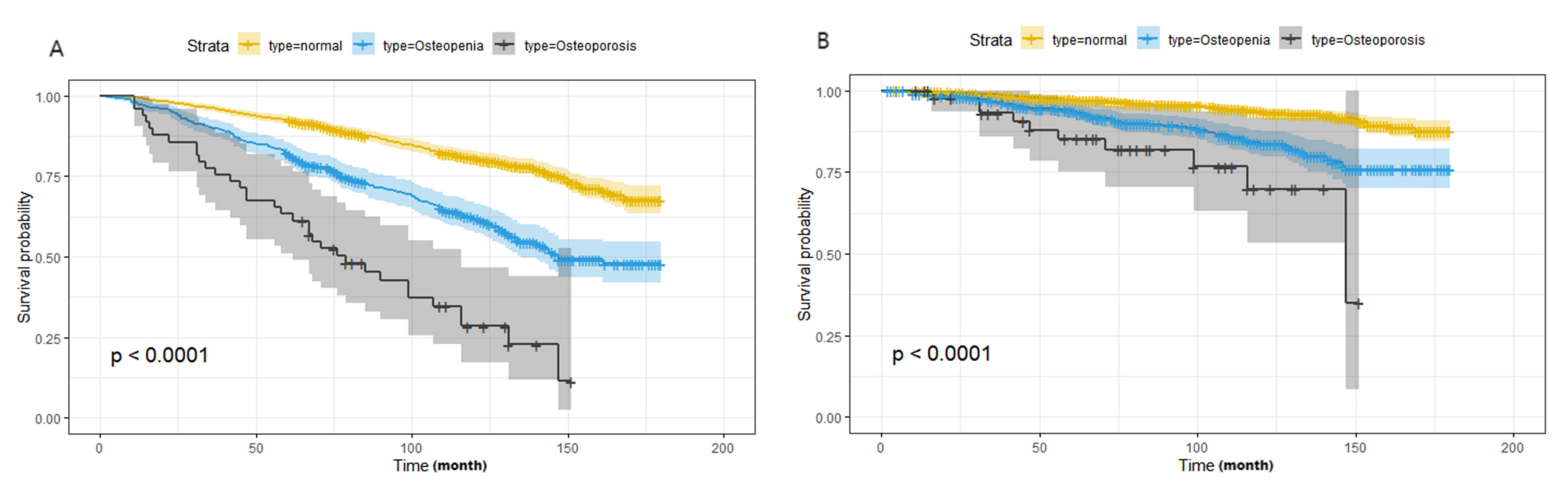
**Kaplan Meier survival analysis for the distribution of all-cause and CVD mortality according to the groups of bone mineral density**. (A) All-cause mortality; (B) CVD mortality. Abbreviations: NHANES, National Health and Nutrition Examination Survey; CVD, cardiovascular disease.

All-cause death and CVD death were taken as the end-point follow-up events for T2DM participants. Kaplan Meier survival analysis showed a statistically significant difference in the incidence of cumulative end events among the normal bone mass group, osteopenia group and osteoporosis group (Log-rank test, *p* < 0.0001) (Fig. 3).

Table 2 displayed the correlations between BMD value and both all-cause mortality and CVD mortality. Osteopenia and osteoporosis showed a significant connection with CVD mortality and all-cause mortality, with the mortality HR (95% CI) at 1.44 (1.00, 2.07) and 2.54 (1.12, 5.75) for CVD mortality, and 1.14 (1.18, 1.67) and 3.33 (2.02, 5.50) for all-cause mortality, respectively, when compared to normal bone mass in the fully adjusted model 3. A statistically significant correlation was found between BMD and mortality from CVD as well as overall mortality. An increase of 1 unit in the natural log-transformed total femur BMD value was associated with a 73% lower risk of CVD mortality and a 65% lower risk of all-cause mortality in model 3; while a 1-unit increase in the natural log-transformed femur neck BMD value was linked to a 78% lower risk of CVD mortality and a 56% lower risk of all-cause mortality in model 3. Similar results were seen in the subgroup analysis (Table 3), with a trend of higher CVD mortality and overall mortality in individuals with T2DM and worsening bone density loss.

**Table 2.** **Hazard ratios (95% CI) for risk of CVD and all-cause mortality according to the groups of bone mineral density**.

	Model 1		Model 2		Model 3	
	HR^1^	*p*	HR^1^	*p*	HR^1^	*p*
	(95% CI)^1^		(95% CI)^1^		(95% CI)^1^	
CVD Mortality						
Normal	Reference	-	Reference	-	Reference	-
Osteopenia	1.57 (1.14, 2.15)	0.006	1.54 (1.12, 2.12)	0.008	1.44 (1.00, 2.07)	0.048
Osteoporosis	2.62 (1.28, 5.40)	0.009	2.53 (1.19, 5.36)	0.016	2.54 (1.12, 5.75)	0.025
Per 1 unit higher in the Total femur BMD	0.19 (0.06, 0.55)	0.002	0.22 (0.07, 0.64)	0.006	0.27 (0.08, 0.90)	0.033
Per 1 unit higher in the Femur neck BMD	0.16 (0.04, 0.64)	0.010	0.17 (0.04, 0.73)	0.017	0.22 (0.05, 1.01)	0.051
*p* for trend	*p* = 0.009		*p* = 0.015		*p* = 0.025	
All-cause Mortality						
Normal	Reference	-	Reference	-	Reference	-
Osteopenia	1.50 (1.24, 1.83)	<0.001	1.47 (1.22, 1.78)	<0.001	1.14 (1.18, 1.67)	<0.001
Osteoporosis	3.23 (1.98, 5.27)	<0.001	3.05 (1.90, 4.91)	<0.001	3.33 (2.02, 5.50)	<0.001
Per 1 unit higher in the Total femur BMD	0.23 (0.11, 0.51)	<0.001	0.30 (0.13, 0.68)	0.004	0.35 (0.16, 0.77)	0.010
Per 1 unit higher in the Femur neck BMD	0.23 (0.14, 0.70)	0.005	0.36 (0.16, 0.83)	0.017	0.44 (0.20, 0.97)	0.042
*p* for trend	*p* < 0.001		*p* < 0.001		*p* < 0.001	

^1^HR, hazard ratio; CI, confidence interval. Abbreviations: CVD, cardiovascular disease; HDL-C, high density lipoprotein cholesterol; TC, total cholesterol; TG, total triglyceride; Cre, creatinine; BUN, blood urea nitrogen; UA, uric acid; BMD, bone mineral density; GED, general educational development; Grad, graduate. Model 1: age (continuous), gender (male or female), and race (Non-Hispanic White, Non-Hispanic Black, Mexican American, Other Hispanic, Other/multiracial) were adjusted. Model 2: age (continuous), gender (male or female), race and ethnicity (Non-Hispanic White, Non-Hispanic Black, Mexican American, Other Hispanic, Other/multiracial), Education attainment (Less Than 9th Grade, 9–11th Grade, High School Grad/GED, Some College or AA degree, College Graduate or above), Alcohol consumption (1–5 drinks/month, 5–10 drinks/month, 10+ drinks/month, Non-drinker), Smoking status (Current smoker, Former smoker, Never smoker) and Physical activity (High physical activity, Low physical activity, No physical activity) were adjusted. Model 3: age (continuous), gender (male or female), race and ethnicity (Non-Hispanic White, Non-Hispanic Black, Mexican American, Other Hispanic, Other/multiracial), Education attainment (Less Than 9th Grade, 9–11th Grade, High School Grad/GED, Some College or AA degree, College Graduate or above), Alcohol consumption (1–5 drinks/month, 5–10 drinks/month, 10+ drinks/month, Non-drinker), Smoking status (Current smoker, Former smoker, Never smoker) Physical activity (High physical activity, Low physical activity, No physical activity), Waist, HDL-C, TC, TG, Cre, BUN, UA, Total serum calcium and Cotinine (all continuous) were adjusted.

**Table 3.** **Hazard ratios (95% CI) for risk of CVD and all-cause mortality in various subgroups according to the groups of bone mineral density**.

Subgroup		HR (95% CI) for all-cause mortality				HR (95% CI) for CVD mortality			
		Normal	Osteopenia	Osteoporosis	*p* for interaction	Normal	Osteopenia	Osteoporosis	*p* for interaction
Gender					0.1047				0.1877
	Female	1.00	1.73 (1.24, 2.43)	3.97 (2.30, 6.85)		1.00	1.96 (1.09, 3.52)	4.55 (1.29, 16.00)	
	Male	1.00	1.63 (1.19, 2.22)	6.70 (2.29, 19.6)		1.00	1.66 (1.03, 2.68)	1.91 (0.26, 14.00)	
BMI [kg/m^2^]					0.0094				0.0666
	<30	1.00	1.62 (1.26, 2.09)	3.79 (2.08, 2.90)		1.00	1.97 (1.21, 3.21)	3.69 (1.54, 8.55)	
	≥30	1.00	1.65 (1.22, 2.23)	6.95 (2.66, 18.20)		1.00	1.44 (0.76, 2.71)	4.26 (1.45, 8.12)	
Race and ethnicity					0.9487				0.8582
	Non-Hispanic White	1.00	1.59 (1.28, 1.96)	5.07 (2.63, 9.79)		1.00	1.53 (1.06, 2.20)	4.21 (1.58, 11.20)	
	Other	1.00	1.92 (1.31, 2.80)	3.81 (2.12, 6.85)		1.00	2.24 (1.22, 4.10)	2.38 (0.75, 7.50)	
Education attainment					0.7360				0.4267
	High School Grad or below	1.00	1.74 (1.34, 2.25)	3.12 (1.73, 5.62)		1.00	1.61 (1.11, 2.33)	2.83 (0.89, 9.00)	
	College Graduate or above	1.00	1.51 (1.04, 2.19)	9.86 (4.34, 22.40)		1.00	2.07 (1.19, 3.60)	5.59 (1.99, 15.70)	
Alcohol consumption					0.9383				0.8202
	Drinker	1.00	1.79 (1.37, 2.32)	3.95 (1.75, 8.93)		1.00	1.51 (1.01, 2.24)	2.25 (1.13, 4.48)	
	Non-drinker	1.00	1.33 (0.87, 2.04)	4.57 (2.28, 9.13)		1.00	2.25 (1.13, 4.48)	5.92 (1.75, 20.00)	
Smoking status					0.5423				0.2308
	Current/Former smoker	1.00	1.76 (1.44, 2.16)	3.80 (2.07, 7.00)		1.00	1.72 (1.17, 2.54)	2.37 (0.85, 6.58)	
	Never smoker	1.00	1.37 (0.97, 1.94)	5.40 (2.68, 10.09)		1.00	1.67 (0.97, 2.91)	5.49 (2.17, 14.90)	
Physical activity					0.5616				0.2567
	Physical activity	1.00	1.58 (1.09, 2.29)	2.67 (0.78, 9.22)		1.00	1.80 (0.96, 3.36)	0.74 (0.11, 5.10)	
	No physical activity	1.00	1.65 (1.26, 2.14)	4.87 (2.85, 8.34)		1.00	1.50 (1.00, 2.25)	4.04 (1.70, 9.62)	
Waist [cm]					0.0038				0.0218
	<108	1.00	1.54 (1.10, 2.14)	2.98 (0.77, 11.60)		1.00	1.46 (0.70, 3.02)	3.01 (1.11, 7.23)	
	≥108	1.00	1.74 (1.29, 2.36)	3.87 (2.08, 7.20)		1.00	1.91 (1.14, 3.19)	3.28 (1.26, 8.52)	
HDL-C [mg/dL]					0.5884				0.4192
	<179	1.00	1.75 (1.25, 2.44)	4.15 (1.73, 9.93)		1.00	1.66 (1.04, 2.63)	1.84 (0.46, 7.31)	
	≥179	1.00	1.55 (1.15, 2.09)	4.16 (2.42, 7.17)		1.00	1.81 (1.04, 3.15)	4.57 (1.57, 13.30)	
TC [mg/dL]					0.0315				0.7192
	<182	1.00	1.89 (1.49, 2.40)	4.12 (2.15, 7.91)		1.00	1.80 (1.15, 2.83)	3.10 (1.13, 8.51)	
	≥182	1.00	1.42 (0.99, 2.03)	5.16 (2.40, 11.10)		1.00	1.55 (0.87, 2.76)	4.74 (1.21, 18.50)	
TG [mg/dL]					0.0993				0.0171
	<157	1.00	1.83 (1.39, 2.42)	3.05 (1.72, 5.41)		1.00	1.86 (1.19, 2.90)	3.09 (1.20, 7.98)	
	≥157	1.00	1.52 (1.14, 2.04)	7.94 (4.37, 14.40)		1.00	1.50 (0.94, 2.41)	3.83 (1.13, 13.00)	

All the models were adjusted for age, gender, race and ethnicity, Education attainment, Alcohol consumption, Smoking status, Physical activity, Waist, HDL-C, TC, TG, Cre, BUN, UA, Total serum calcium and Cotinine, with exception of stratifying factors. Abbreviations: HR, hazard ratio; CI, confidence interval; HDL-C, high density lipoprotein cholesterol; TC, total cholesterol; TG, total triglyceride; Grad, graduate; BMI, body mass index; Cre, creatinine; BUN, blood urea nitrogen; UA, uric acid.

### 3.3 Stratified and Sensitivity Analyses

Multiple stratified analyses were performed. After adjusting for potential risk factors, we found that the association between BMD value and CVD mortality was modified by the waist. Osteoporosis was linked to a higher chance of CVD mortality in individuals with larger waist measurements, with a hazard ratio of 3.28 (95% CI = 1.26, 8.52; *p* = 0.0218 for interaction). Participants with elevated total TG levels had a 3.83 times higher risk of CVD mortality when also diagnosed with osteoporosis (95% CI = 1.13, 13.00; *p* for interaction = 0.0171) (Table 3). In order to investigate the impact of reverse causation, the analysis excluded 65 participants who passed away within the initial 2 years of the study. The results from the remaining sample were comparable to those seen in the complete sample (**Supplementary Table 2**). We further excluded 517 participants with a history of CVD at baseline and showed no substantial change in the risk of CVD mortality in participants with osteoporosis under all models; however, participants with reduced bone mass had a statistically insignificant result compared to those with normal bone mass, although the risk of CVD mortality was increased (*p* > 0.05) (**Supplementary Table 3**).

## 4. Discussion

Analyzing data from NHANES 2005–2010 and 2013–2014, we found a strong correlation between higher BMD and lower mortality rates for both all causes and CVDs in American adults diagnosed with T2DM. Furthermore, our research revealed that osteoporosis and osteopenia were linked to a higher risk of all-cause and CVD mortality in T2DM patients during long-term monitoring, even after adjusting for multiple variables, and stratified analysis in relation to mortality from CVD. Prior research has indicated that the association between osteopenia and osteoporosis and the risk of all-cause mortality and CVD mortality is still a topic of debate. According to the study by Cai S *et al*. [21], individuals with osteoporosis in various parts of the body such as the total femur, femur neck, and intertrochanter had a greater risk of all-cause mortality compared to those without osteoporosis. Significant L-shaped relationships were found only for mortality in heart disease with BMD increments found within specific femur limits, but these relationships disappeared as BMD continued to rise [21]. Additional results from cohorts representing the entire nation also indicated that osteoporosis was linked to a higher chance of death from any cause (HR = 1.37, 95% CI = 1.11–1.68), particularly among older individuals with lower BMI. There was no significant association between osteoporosis and CVD mortality, which could be attributed to the limited number of CVD and cancer deaths, as well as the short duration of follow-up [22]. Other studies have found some positive associations on the relationship between osteoporosis and CVD risk. The study by Rodríguez-Gómez *et al*. [23], which included a total of 305,072 UK Biobank participants diagnosed with osteoporosis at baseline, found that men with osteoporosis had a higher mortality risk from CVD (HR = 1.68, 95% CI = 1.19–2.37). However, women with osteoporosis only had a higher risk of incident CVD (HR = 1.24, 95% CI = 1.97–1.44), and the risk of CVD mortality was not affected [23]. Calcaneal quantitative ultrasound (QUS) was utilized to assess bone material properties, particularly in elderly women, and could also be used to diagnose osteoporosis. In a prospective study of aged women, Gebre *et al*. [24] discovered that quantitative ultrasound measurements of the calcaneus were independently associated with increased cardiovascular and all-cause mortality, regardless of established cardiovascular risk factors. Reducing broadband ultrasound attenuation (BUA) in the minimally and multivariable adjusted model including cardiovascular risk factors increased the relative hazard for all-cause mortality (HR = 1.15, 95% CI = 1.06–1.261) and CVD mortality (HR = 1.20, 95% CI = 1.04–1.38). Nonetheless, this observational study was limited to only geriatric females, and the results were not derived from the analysis of BMD values [24]. In a similar study called the AA-DHS, vertebral BMD (vBMD) was analyzed in 675 African American men with T2DM and the results suggested that lower vBMD was associated with increased all-cause mortality [25]. However, this study also showed that lower vBMD was not linked to other mortality risk factors, including subclinical atherosclerosis [25]. In light of this, we embarked on a comprehensive study to determine the relationship between BMD and all-cause mortality and CVD mortality across multiple anatomical sites. Our investigation showed the independent and incremental value of low BMD, particularly with osteoporosis in assessing the risk of CVD in adults with T2DM. Our findings in T2DM contribute to the existing literature on this subject.

Diabetes mellitus has been linked to impaired bone quality and increased risk of fracture [26]. The pathophysiology of osteoporosis caused by T2DM is multifactorial, involving reduced bone formation, osteoblast dysfunction, and low bone turnover [27]. Hyperlipidemia, impaired insulin signaling, low levels of insulin-like growth factor 1, reactive oxygen species generation, and inflammation are all linked to diabetes and hyperglycemia, and may all contribute to the inhibition of osteoblast activity [28, 29, 30, 31]. Degraded bone quality and microarchitectural defects are the result of multiple factors, including chronic hyperglycemia and skeletal advanced glycation end products (AGES), which irreversibly accumulate from the nonenzymatic addition of sugar moieties to the amine groups of proteins. AGES negatively impacted skeletal integrity, particularly type 1 collagen [32, 33, 34, 35, 36, 37]. T2DM and osteoporosis are two extremely common adverse conditions of CVD, and share several risk factors involved in their pathophysiological mechanisms, including aging, smoking, weight gain, inactivity, estrogen deficiency, hyperlipidemia, oxidative stress, chronic inflammation and common polygenes [38, 39, 40, 41, 42]. Therefore, an increase in bone loss in this particular T2DM population is strongly associated with an increase in the risk of cardiovascular and all-cause mortality.

It is worth noting that in the sensitivity analysis, when we only retained T2DM patients with no prior history of acute CVD, such as heart failure, angina pectoris, coronary heart disease, myocardial infarction, and stroke, the increased risk of CVD mortality in T2DM patients with reduced bone mass was not significant compared to normal bone mass patients, but the increased risk of CVD mortality in T2DM patients with osteoporosis remained stable. This suggests that the increased risk of mortality directly caused by developing CVD was only significant when bone mineral content was extremely low, and it also confirmed that osteoporosis was closely associated to both CVD incidence and CVD mortality risk. Due to the limitations of the NHANES database, specific analysis of HbA1c levels in T2DM patients was not conducted in this study, which may affect the accuracy of the study results. However, in a retrospective cross-sectional study involving 856 male T2DM patients, HbA1c and insulin use did not differ significantly between CVD and non-CVD individuals [43], and another study concluded that age-adjusted HbA1c was not a risk factor for osteoporosis in postmenopausal women with T2DM [44]. In addition, this study did not evaluate parameters related to calcium metabolism disorders such as blood phosphorus, vitamin D, and parathyroid hormone, which are potential mediators between osteoporosis and CVDs. Only total blood calcium was included in the confounding factors for correction, which may make the study results one-sided.

In stratified analyses, our study found that the independent associations of osteoporosis and osteopenia with an increased risk of CVD mortality were stronger in participants with wider waist and higher TG levels, possibly because the wide waist and high TG levels were themselves independent risk factors for adverse cardiovascular outcomes in T2DM. Our results showed that patients with osteopenia and osteoporosis, in addition to research by Yang and Shen [45], found a link between BMI and lumbar vertebral and femoral neck BMD. Zhao *et al*. [46] found a favorable correlation between HDL-C levels and BMD, as well as a preventive impact against osteoporosis (OR = 0.07, 95% CI = 0.01, 0.53, *p* < 0.05). This was consistent with our findings. However, the link between TC and TG levels and osteoporosis remained inconclusive. In a previous study, low BMD was associated with atherosclerotic lipid abnormalities [47], but this association was not consistently seen in other studies. A recent meta-analysis suggested that among subjects who were not taking lipid-lowering drugs, TC and TG in osteopenia were not significantly increased or decreased [48]. These factors were generally considered to be protective factors for CVD, which was exactly the opposite of our findings. To explain this phenomenon, we believe that diabetes-induced osteoporosis represents the combined impact of conventional osteoporosis with the additional fracture burden attributed to diabetes, not just the impaired bone quality. T2DM patients had a significantly increased risk of fracture, as well as an increased risk of complications following fracture, such as delayed wound closure, infectious, and peri-operative cardiovascular events [49, 50]. Therefore, this might be the reason why even though there were more cardiovascular protective factors in T2DM patients with osteoporosis, the risk of all-cause mortality and CVD mortality remained high.

## 5. Study Strengths and Limitations

In recent years, there has been a rapid rise in the occurrence of osteoporosis as a result of T2DM. However, the correlation between BMD or osteoporosis and the likelihood of all-cause mortality and CVD mortality has not been extensively researched in this specific population group. Specifically, there were no conclusive findings regarding the associations with the risk of mortality from CVD. Our research validated a notable inverse relationship between BMD and the likelihood of death from any cause and CVD in individuals with T2DM, with both osteoporosis and osteopenia independently linked to higher risks of all-cause mortality and CVD mortality. These findings expanded the range of potential applications of the previous results and confirmed that osteoporosis was a separate indicator of CVD mortality.

This study also has some limitations. First, the cross-sectional nature of this study made it impossible to measure changes in BMD over time, so we could not determine whether their changes were related to these associations, so that the evaluation of causality in osteoporosis and T2DM was also limited. Second, most of the studies examining the association between bone health and cardiovascular health had used postmenopausal female populations who had a relatively higher risk for both osteoporosis and CVDs when compared with premenopausal women [13, 14]. Although this study performed a weighted analysis, the sample size was relatively small, and the number of postmenopausal women with T2DM and osteoporosis was very small, so we did not conduct further sensitivity analysis on this population. Therefore, further prospective studies with larger sample sizes are required. Third, some data on variables was obtained through self-reported questionnaires, which might introduce recollection bias and under-represent the real situation. In addition, we also may not have accounted for all potential confounders, even after controlling for recognized risk factors for mortality.

## 6. Conclusions

In conclusion, our findings indicated that osteoporosis and osteopenia were independently linked with an elevated risk of all-cause and CVD mortality among US individuals with T2DM at multifactor-adjusted long-term follow-up. These associations were stronger in participants with larger waists and higher total TG levels. As a result, the clinical diagnosis of osteopenia or osteoporosis in adults with T2DM provided independent prognostic value for CVD and all-cause mortality. Future research should explore the potential mechanisms of this association and examine whether therapies aimed at improving BMD can reduce the incidence of CVD events in this population.

## Data Availability

The datasets generated and analyzed during the current study are available from the corresponding author on reasonable request.

## Abbreviations

BMD, bone mineral density; T2DM, type 2 Diabetes Mellitus; CVD, cardiovascular disease; NHANES, National Health and Nutrition Examination Survey; BMI, body mass index; HDL-C, high density lipoprotein cholesterol; TC, total cholesterol; TG, total triglyceride; Cre, creatinine; BUN, blood urea nitrogen; UA, uric acid; HbA1c, hemoglobin A1c; DXA, dual-energy X-ray absorptiometry; QUS, quantitative ultrasound; BUA, broadband ultrasound attenuation; AGES, advanced glycation end products; RCS, restricted cubic spline; HR, hazard ratio; CI, confidence interval.

## Supplementary Material

Supplementary material associated with this article can be found, in the online version, at https://doi.org/10.31083/j.rcm2512434.
