# Supplementary material for: Bone Mineral Density is Negatively Associated with Risk of All-Cause and Cardiovascular Mortality among Adults with Type 2 Diabetes Mellitus: A Cross-sectional Study of the NHANES 2005–2010, 2013–2014
Source: Rev Cardiovasc Med. 2024 Dec 11;25(12):434. doi: 10.31083/j.rcm2512434 (PMC11683720; doi:10.31083/j.rcm2512434)
Supplement: Supplementary file 1 [file 2153-8174-25-12-434-s1.docx]

|  | **Osteopenia** | **Osteoporosis** |
| --- | --- | --- |
| Male |  |  |
| Total femur BMD (g/cm^2^) | 0.68–0.90 | < 0.68 |
| Femur neck BMD (g/cm^2^) | 0.59–0.79 | < 0.59 |
| Female |  |  |
| Total femur BMD (g/cm^2^) | 0.64–0.82 | < 0.64 |
| Femur neck BMD (g/cm^2^) | 0.56–0.74 | < 0.56 |

Abbreviations:BMD, bone mineral density.

Supplementary Table 1: Definition of osteoporosis and osteopenia.

|  | **Model 1** | | **Model 2** | | **Model 3** | |
| --- | --- | --- | --- | --- | --- | --- |
|  | **HR^1^**  **(95% CI)^1^** | ***p*** | **HR^1^**  **(95% CI)^1^** | ***p*** | **HR^1^**  **(95% CI)^1^** | ***p*** |
| **CVD mortality** | | | | | | |
| Normal | Ref | - | Ref | - | Ref | - |
| Osteopenia | 1.49(1.06,2.08) | **0.020** | 1.44(1.02,2.01) | **0.036** | 1.35(0.92,2.00) | 0.130 |
| Osteoporosis | 2.74(1.29,5.82) | **0.009** | 2.59(1.18,5.71) | **0.018** | 2.64(1.11,6.25) | **0.028** |
| Per 1 unit higher in the Total femur BMD | 0.21(0.07,0.64) | **0.006** | 0.25(0.08,0.77) | **0.016** | 0.29(0.08,1.03) | 0.055 |
| Per 1 unit higher in the Femur neck BMD | 0.16(0.03,0.71) | **0.016** | 0.18(0.04,0.83) | **0.029** | 0.21(0.04,1.10) | 0.065 |
| ***P* for trend** | ***P=*0.009** | | ***P=*0.018** | | ***P=*0.028** | |
| **All-cause Mortality** | | | | | | |
| Normal | Ref | - | Ref | - | Ref | - |
| Osteopenia | 1.47(1.19,1.81) | **<0.001** | 1.44(1.17,1.77) | **<0.001** | 1.43(1.19,1.71) | **<0.001** |
| Osteoporosis | 3.04(1.79,5.14) | **<0.001** | 2.90(1.72,4.89) | **<0.001** | 3.38(1.94,5.88) | **<0.001** |
| Per 1 unit higher in the Total femur BMD | 0.29(0.13,0.64) | **0.002** | 0.36(0.15,0.85) | **0.019** | 0.35(0.16,0.76) | **0.009** |
| Per 1 unit higher in the Femur neck BMD | 0.36(0.16,0.80) | **0.012** | 0.41(0.18,0.94) | **0.035** | 0.41(0.18,0.93) | **0.033** |
| ***P* for trend** | ***P*<0.001** | | ***P*<0.001** | | ***P*<0.001** | |
| ^1^HR=Hazard Ratio,CI=confidence Interval  Abbreviations: CVD, cardiovascular disease; BMI, body mass index;HDL-C,high density lipoprotein cholesterol;TC,total cholesterol;TG,total triglyceride;Cre,creatinine;BUN,blood urea nitrogen;UA,uric acid;BMD,Bone mineral density;NHANES, National Health and Nutrition Examination Survey.  Model 1: age (continuous), gender (male or female), and race (Non-Hispanic White,  Non-Hispanic Black, Mexican American, Other Hispanic,Other/multiracial) were adjusted.  Model 2: age(continuous) , gender (male or female), race and ethnicity (Non-Hispanic White,  Non-Hispanic Black, Mexican American, Other Hispanic,Other/multiracial) ,Education attainment(Less Than 9th Grade,9-11th Grade,High School Grad/GED,Some College or AA degree,College Graduate or above),Alcohol consumption(1-5 drinks/month,5-10 drinks/month,10+ drinks/month,Non-drinker),Smoking status(Current smoker,Former smoker,Never smoker) and Physical activity(High physical activity,Low physical activity,No physical activity) were adjusted.  Model 3:age(continuous) , gender (male or female), race and ethnicity (Non-Hispanic White,  Non-Hispanic Black, Mexican American, Other Hispanic,Other/multiracial) ,Education attainment(Less Than 9th Grade,9-11th Grade,High School Grad/GED,Some College or AA degree,College Graduate or above),Alcohol consumption(1-5 drinks/month,5-10 drinks/month,10+ drinks/month,Non-drinker),Smoking status(Current smoker,Former smoker,Never smoker) Physical activity(High physical activity,Low physical activity,No physical activity),Waist, HDL-C,TC,TG,Cre,BUN,UA,Total serum calcium and Cotinine (all continuous) were adjusted.  Supplementary Table 2:Hazard Ratios (95% CI) for risk of CVD and all-cause mortality according to the groups of bone mineral density.Participants who died during the first two years of follow-up were excluded. | | | | | | |

|  | **Model 1** | | **Model 2** | | **Model 3** | |
| --- | --- | --- | --- | --- | --- | --- |
|  | **HR^1^**  **(95% CI)^1^** | ***p*** | **HR^1^**  **(95% CI)^1^** | ***p*** | **HR^1^**  **(95% CI)^1^** | ***p*** |
| **CVD mortality** | | | | | | |
| Normal | Ref | - | Ref | - | Ref | - |
| Osteopenia | 1.38(0.86,2.23) | 0.2 | 1.35(0.81,2.24) | 0.2 | 1.11(0.66,1.85) | 0.7 |
| Osteoporosis | 4.93(2.28,10.6) | **<0.001** | 4.88(2.34,10.2) | **<0.001** | 3.69(1.49,9.16) | **0.005** |
| Per 1 unit higher in the Total femur BMD | 0.12(0.02,0.64) | **0.012** | 0.13(0.03,0.66) | **0.014** | 0.28(0.07,1.12) | 0.072 |
| Per 1 unit higher in the Femur neck BMD | 0.08(0.01,0.80) | **0.032** | 0.08(0.01,0.84) | **0.035** | 0.13(0.02,1.01) | 0.051 |
| ***P* for trend** | ***P*<0.001** | | ***P*<0.001** | | ***P=*0.005** | |
| **All-cause Mortality** | | | | | | |
| Normal | Ref | - | Ref | - | Ref | - |
| Osteopenia | 1.17(0.86,1.60) | 0.3 | 1.21(0.88,1.67) | 0.2 | 1.16(0.86,1.57) | 0.3 |
| Osteoporosis | 3.58(2.02,6.35) | **<0.001** | 3.73(2.16,6.34) | **<0.001** | 3.81(2.04,7.09) | **<0.001** |
| Per 1 unit higher in the Total femur BMD | 0.36(0.12,1.05) | 0.061 | 0.37(0.12,1.16) | 0.088 | 0.44(0.18,1.10) | 0.079 |
| Per 1 unit higher in the Femur neck BMD | 0.52(0.17,1.60) | 0.3 | 0.52(0.16,1.71) | 0.3 | 0.56(0.20,1.59) | 0.3 |
| ***P* for trend** | ***P*<0.001** | | ***P*<0.001** | | ***P*<0.001** | |
| ^1^HR=Hazard Ratio,CI=confidence Interval  Abbreviations: CVD, cardiovascular disease; BMI, body mass index;HDL-C,high density lipoprotein cholesterol;TC,total cholesterol;TG,total triglyceride;Cre,creatinine;BUN,blood urea nitrogen;UA,uric acid;BMD,Bone mineral density;NHANES, National Health and Nutrition Examination Survey.  Model 1: age (continuous), gender (male or female), and race (Non-Hispanic White,  Non-Hispanic Black, Mexican American, Other Hispanic,Other/multiracial) were adjusted.  Model 2: age(continuous) , gender (male or female), race and ethnicity (Non-Hispanic White,  Non-Hispanic Black, Mexican American, Other Hispanic,Other/multiracial) ,Education attainment(Less Than 9th Grade,9-11th Grade,High School Grad/GED,Some College or AA degree,College Graduate or above),Alcohol consumption(1-5 drinks/month,5-10 drinks/month,10+ drinks/month,Non-drinker),Smoking status(Current smoker,Former smoker,Never smoker) and Physical activity(High physical activity,Low physical activity,No physical activity) were adjusted.  Model 3:age(continuous) , gender (male or female), race and ethnicity (Non-Hispanic White,  Non-Hispanic Black, Mexican American, Other Hispanic,Other/multiracial) ,Education attainment(Less Than 9th Grade,9-11th Grade,High School Grad/GED,Some College or AA degree,College Graduate or above),Alcohol consumption(1-5 drinks/month,5-10 drinks/month,10+ drinks/month,Non-drinker),Smoking status(Current smoker,Former smoker,Never smoker) Physical activity(High physical activity,Low physical activity,No physical activity),Waist, HDL-C,TC,TG,Cre,BUN,UA,Total serum calcium and Cotinine (all continuous) were adjusted.  Supplementary Table 3:Hazard Ratios (95% CI) for risk of CVD and all-cause mortality according to the groups of bone mineral density.Participants with a history of cardiovascular disease at baseline were excluded. | | | | | | |
